# Accuracy and Interobserver Reliability of Preoperative Templating of the Tibial Component in Medial Unicompartmental Knee Replacement

**DOI:** 10.7759/cureus.112476

**Published:** 2026-07-11

**Authors:** Mohamed Mashaly, Raja Babar Akram, Harry Krishnan

**Affiliations:** 1 Trauma and Orthopaedics, South Warwickshire University NHS Foundation Trust, Warwick, GBR

**Keywords:** component sizing, interobserver reliability, preoperative templating, traumacad, ukr, unicompartmental knee replacement

## Abstract

Background

Preoperative templating is an important component of surgical planning in knee arthroplasty. In medial unicompartmental knee replacement (UKR), accurate tibial component sizing can be challenging due to limited surgical exposure and the risk of posterior cortical injury. This study aimed to evaluate the accuracy and interobserver reliability of preoperative templating using templating software in predicting tibial component size.

Methods

A retrospective observational study was conducted including 69 patients who had undergone medial UKR between January 2023 and December 2024. Two orthopaedic specialty doctors independently templated preoperative lateral knee radiographs using TraumaCad software (Brainlab, Munich, Germany), blinded to the actual implanted component size and to each other's assessments. Predicted sizes were compared between observers and against the implanted components. Interobserver reliability was assessed using the intraclass correlation coefficient (ICC) with a two-way random effects model for absolute agreement and weighted Cohen's kappa with quadratic weights.

Results

Exact agreement between observers was observed in 39 of 69 cases (56.5%), increasing to 68 of 69 (98.6%) within ±1 size. Observer 1 predicted the exact implanted size in 35 cases (50.7%), improving to 60 cases (87.0%) within ±1 size. Observer 2 demonstrated higher accuracy with exact prediction in 42 cases (60.9%) and 66 cases (95.7%) within ±1 size. Interobserver reliability was almost perfect with an ICC of 0.82 (95% CI: 0.72-0.88) and a weighted kappa value of 0.82. Bland-Altman analysis revealed minimal systematic bias between observers (mean difference 0.33 sizes). Observer 1 showed systematic oversizing compared to actual implants (mean bias +0.45 sizes, p<0.001), while Observer 2 demonstrated no significant bias (mean bias +0.12 sizes, p=0.19).

Conclusion

Preoperative templating using TraumaCad demonstrated almost perfect interobserver reliability and good-to-excellent accuracy within a clinically acceptable range. It represents a useful adjunct in surgical planning for medial UKR. Future studies comparing templating performance across different software platforms would be valuable.

## Introduction

Unicompartmental knee replacement (UKR) is an established treatment for isolated compartment osteoarthritis, offering advantages over total knee replacement including faster recovery, preservation of native knee kinematics, and improved functional outcomes [1,2]. However, it is technically demanding, with limited surgical exposure and reduced margin for error.

Accurate tibial component sizing is critical in UKR. Undersizing may lead to inadequate cortical support, implant subsidence, and increased risk of periprosthetic fracture [3,4]. Conversely, oversizing may result in cortical overhang causing soft tissue irritation and medial collateral ligament impingement [5]. Additionally, the tibial cut must be performed carefully to avoid posterior cortical breach, particularly given the restricted visualization inherent to the procedure.

Preoperative templating allows surgeons to anticipate implant size, plan surgical steps, and ensure appropriate implant availability. Previous studies on total knee replacement have demonstrated that digital templating is generally accurate within one size and may improve intraoperative decision-making [6-8]. Studies have reported exact match rates of 48%-71% for total knee replacement components, with accuracy improving to 93% within ±1 size [6,9]. However, evidence specific to UKR remains limited, and few studies have evaluated the interobserver reliability of templating methods [10,11].

This study evaluates the accuracy and interobserver reliability of preoperative templating using TraumaCad software (Brainlab, Munich, Germany) for tibial component sizing in medial UKR.

## Materials and methods

Study design and setting

A retrospective observational study was conducted at the Department of Orthopaedics, South Warwickshire University NHS Foundation Trust, Warwickshire, Great Britain, between January 2023 and December 2024. This study was a retrospective audit of anonymised radiographic and surgical data. Ethical approval was not required as per the institutional policy. All data were handled in accordance with NHS information governance policies and the UK General Data Protection Regulation (GDPR).

Inclusion and exclusion criteria

Patients undergoing primary medial UKR with available preoperative lateral knee radiographs and complete operative records documenting implanted component size were included. Revision procedures, cases with poor-quality radiographs precluding accurate templating, or those with missing implant data were excluded.

Templating method

Templating was performed using TraumaCad software, version 3.5. Two experienced orthopaedic specialty doctors (Observer 1 and Observer 2) independently templated all cases using preoperative lateral knee radiographs. All procedures were performed using the Physica ZUK (Enovis/LimaCorporate, Udine, Italy), which is a fixed-bearing UKR system. Procedures were performed by multiple consultant orthopaedic surgeons within the department using a standardised surgical technique, reflecting routine clinical practice. Templating was performed retrospectively on radiographs from previously completed surgical cases. Both observers were blinded to the actual implanted component size and to each other's template selections at the time of assessment. Radiographs were reviewed independently in a random order to minimize recall bias.

No calibration markers were used; a standard magnification factor of 118%, routinely applied in our institution, was used. Each observer selected the tibial component size that best matched the anteroposterior dimension of the proximal tibia on the lateral radiograph, ensuring adequate cortical coverage while avoiding overhang. Template sizes ranged from 1 to 6, corresponding to available implant sizes.

Outcome measures

The primary outcome was interobserver reliability, as establishing reproducibility is fundamental to validating any templating method. Interobserver reliability was assessed using the intraclass correlation coefficient (ICC) with a two-way random effects model for absolute agreement and weighted Cohen's kappa with quadratic weights. Secondary outcomes included exact interobserver agreement (%), agreement within ±1 size (%), accuracy of each observer compared to the actual implanted size, exact match rates (%), accuracy within ±1 size (%), correlation with the implanted size (Pearson correlation coefficient), and systematic sizing bias.

Statistical analysis

Interobserver reliability was assessed using ICC with a two-way random effects model for absolute agreement, with single measures (ICC(2,1)) [12,13]. Weighted Cohen's kappa with quadratic weights was calculated to account for the ordinal nature of sizing data and the clinical relevance of disagreement magnitude [14,15].

Agreement thresholds were defined as poor (<0.50), moderate (0.50-0.75), good (0.75-0.90), and excellent (>0.90). Pearson correlation coefficients were calculated to assess linear relationships. Bland-Altman analysis was performed to evaluate systematic bias and limits of agreement [16]. Systematic bias was tested using the Wilcoxon signed-rank test. Statistical significance was set at p<0.05. Analyses were performed using Python 3.9 with SciPy and Scikit-learn libraries.

## Results

Cohort chartaacteristics

A total of 69 patients met the inclusion criteria and were analyzed. All underwent medial unicompartmental knee replacement with complete preoperative radiographs and operative records.

Implant size distribution

Actual implanted sizes ranged from 1 to 6, with the majority being sizes 3-4, representing 65.2% (45/69) of cases. The complete distribution is presented in Table 1. Observer 1 templated toward larger sizes (63.8% selected sizes 4-6) compared to Observer 2 (66.6% selected sizes 2-4), reflecting different interpretation patterns of the radiographic tibial dimensions.

**Table 1:** Distribution of templated and actual component sizes

Component Size	Observer 1 Template, n (%)	Observer 2 Template, n (%)	Actual Implant, n (%)
1	1 (1.4)	1 (1.4)	2 (2.9)
2	2 (2.9)	10 (14.5)	6 (8.7)
3	15 (21.7)	12 (17.4)	19 (27.5)
4	22 (31.9)	25 (36.2)	26 (37.7)
5	22 (31.9)	16 (23.2)	12 (17.4)
6	7 (10.1)	5 (7.2)	4 (5.8)
Total	69 (100)	69 (100)	69 (100)

Interobserver agreement

**Figure 1:**
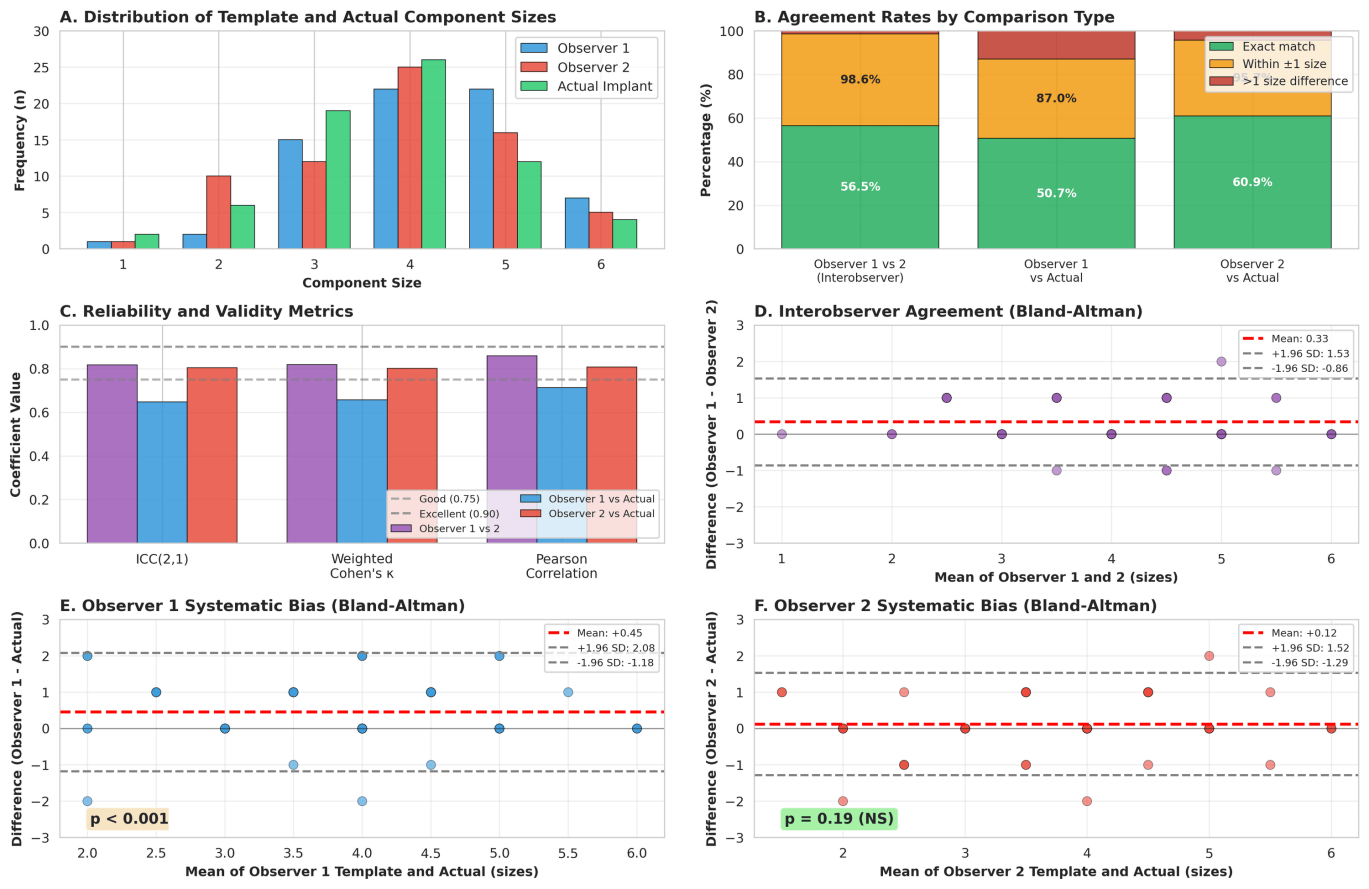
Comprehensive analysis of templating reliability and accuracy Panel A shows the distribution of template and actual component sizes for both observers. Panel B displays agreement rates showing exact match and within ±1 size tolerance. Panel C presents reliability and validity metrics, including ICC, weighted Cohen's kappa, and Pearson correlation coefficients. Panel D shows a Bland-Altman plot demonstrating interobserver agreement with limits of agreement. Panel E shows a Bland-Altman plot demonstrating Observer 1 systematic oversizing bias (mean +0.45 sizes, p<0.001). Panel F shows a Bland-Altman plot demonstrating Observer 2 minimal bias (mean +0.12 sizes, p=0.19, not significant). ICC, intraclass correlation coefficient; NS, not significant The image was created using Python 3 (Python Software Foundation, Wilmington, USA).

Exact agreement between Observer 1 and Observer 2 occurred in 39 of 69 cases (56.5%). When allowing for a ±1 size difference, agreement increased substantially to 68 of 69 cases (98.6%), with only one case showing a difference of 2 sizes (Figures 1A-1B).

Reliability metrics

Interobserver reliability demonstrated good agreement with ICC(2,1) of 0.82 (95% CI: 0.72-0.88) and weighted Cohen's kappa of 0.82 (Table 2; Figure 1C). The Pearson correlation between observers was 0.86 (p<0.001). Bland-Altman analysis revealed a small but statistically significant mean bias of 0.33 sizes (p<0.001) with limits of agreement of -0.86 to +1.53 sizes (Figure 1D).

**Table 2:** Summary of reliability and accuracy metrics ICC, intraclass correlation coefficient

Comparison	Exact Agreement, n (%)	Within ±1 Size, n (%)	ICC (95% CI)	Weighted κ	Pearson r
Interobserver (Observer 1 vs. 2)	39 (56.5)	68 (98.6)	0.82 (0.72-0.88)	0.82	0.86
Observer 1 vs. actual implant	35 (50.7)	60 (87.0)	0.65 (0.49-0.77)	0.66	0.71
Observer 2 vs. actual implant	42 (60.9)	66 (95.7)	0.80 (0.70-0.87)	0.80	0.81

Accuracy - Observer 1

Observer 1 predicted the exact implanted size in 35 of 69 cases (50.7%). Accuracy improved to 60 of 69 cases (87.0%) when allowing for a ±1 size variance. Observer 1 demonstrated substantial validity with an ICC of 0.65 (95% CI: 0.49-0.77), a weighted kappa of 0.66, and a Pearson correlation of 0.71 (p<0.001) (Table 2; Figures 1C, 1E).

Accuracy - Observer 2

Observer 2 showed higher accuracy, predicting the exact implanted size in 42 of 69 cases (60.9%), increasing to 66 of 69 cases (95.7%) within ±1 size. Observer 2 demonstrated good validity with an ICC of 0.80 (95% CI: 0.70-0.87), a weighted kappa of 0.80, and a Pearson correlation of 0.81 (p<0.001) (Table 2; Figures 1C, 1F).

Systematic sizing bias analysis

Bland-Altman analysis revealed a significant systematic oversizing tendency in Observer 1's templates compared to actual implanted components (mean bias +0.45 sizes, p<0.001). Observer 1 templated larger than the actual implant in 30 cases (43.5%), exactly matched in 35 cases (50.7%), and smaller in only 4 cases (5.8%).

In contrast, Observer 2 demonstrated no significant systematic bias (mean bias +0.12 sizes, p=0.19), with a more balanced distribution: larger in 18 cases (26.1%), exact match in 42 cases (60.9%), and smaller in 9 cases (13.0%) (Table 3; Figures 1E-1F).

**Table 3:** Systematic sizing bias analysis

Observer	Mean Bias (sizes)	Templates > Actual, n (%)	Exact Match, n (%)	Templates < Actual, n (%)	Wilcoxon p-value
Observer 1	+0.45	30 (43.5)	35 (50.7)	4 (5.8)	<0.001
Observer 2	+0.12	18 (26.1)	42 (60.9)	9 (13.0)	0.19

## Discussion

This study demonstrates that preoperative templating using TraumaCad software provides almost perfect interobserver reliability (ICC=0.82, κ=0.82) and good to excellent predictive accuracy for tibial component sizing in medial UKR.

Comparison with the existing literature

The findings are consistent with previous works in total knee arthroplasty. Peek et al. reported exact match rates of 60% for tibial and 71% for femoral components in total knee replacement, with all templates accurate within one size [6]. Trickett et al. found 55% accuracy for tibial templating in total knee arthroplasty [9]. Our exact match rates of 51%-61% are comparable, while our within ±1 size accuracy of 87%-96% aligns with the 93% reported in prior studies [6,7].

Few studies have examined the interobserver reliability of knee templating. In hip arthroplasty, The et al. reported ICC values of 0.87-0.95 for digital templating [8], while our ICC of 0.82 demonstrates similarly high reproducibility. The almost perfect interobserver agreement (98.6% within ±1 size) suggests that TraumaCad templating is highly reproducible, which has important implications for training and standardization of preoperative planning [8-10].

Systematic sizing bias and radiographic appearance

A notable finding was the significant oversizing tendency in Observer 1's templates (mean bias +0.45 sizes, p<0.001), while Observer 2 showed no systematic bias (p=0.19). However, despite this systematic difference between observers and actual implant sizes, the interobserver reliability remained almost perfect (ICC=0.82), indicating that both observers applied consistent templating principles [8,10].

Clinical implications

The high interobserver reliability (ICC=0.82) demonstrates that TraumaCad templating produces consistent results across different surgeons. This reproducibility may be particularly valuable in training settings and for standardizing preoperative planning protocols [8,10].

Importantly, 98.6% agreement within ±1 size between observers and 87%-96% accuracy within ±1 size versus actual implants demonstrate clinically acceptable performance. Since most implant systems allow for intraoperative adjustment within adjacent sizes, this margin provides useful surgical planning guidance [6,7]. Templating may assist in anticipating implant size, reducing intraoperative uncertainty, and ensuring availability of appropriate implants, thereby potentially reducing operative time and costs [6,11].

However, templating should be considered an adjunct rather than a substitute for intraoperative assessment. The moderate exact match rates (51%-61%) emphasize that surgical judgment and intraoperative sizing trials remain essential [6,7,11]. The final implant size should always be determined through intraoperative trial sizing and surgical judgment.

Limitations

Several limitations should be acknowledged. First, the retrospective design introduces potential selection bias and prevents prospective validation. Second, as a single-centre study using a single implant system (Physica ZUK), results may not generalize to other institutions or prosthesis designs. Third, procedures were performed by multiple surgeons, which may introduce variability in intraoperative decision-making; however, this also reflects real-world practice and enhances the generalisability of the findings. Fourth, radiographic positioning variations (rotation, flexion angle) were not standardized or controlled (but all were within acceptable limits), which may have affected apparent tibial dimensions. Fifth, we did not correlate templating accuracy with clinical outcomes such as pain scores, function, revision rates, or radiographic evidence of implant loosening. Sixth, we did not evaluate patient-specific factors, such as body mass index, bone quality, severity of deformity, or degree of cartilage loss, that might influence templating accuracy. Finally, the sample size, while adequate for reliability assessment, may limit the detection of subtle factors affecting templating accuracy.

Future directions

Future research should include prospective studies with larger, multicentre cohorts to validate these findings across different populations and implant systems. Investigating the correlation between templating accuracy and postoperative radiographic outcomes (e.g., cortical coverage, overhang) and clinical outcomes would strengthen the evidence base. Evaluation of calibration markers and standardized radiographic positioning protocols may improve templating accuracy. Additionally, studies comparing templating performance across different software platforms would be valuable for optimizing preoperative planning workflows.

## Conclusions

It can be concluded that preoperative templating using TraumaCad software demonstrates almost perfect interobserver reliability (ICC=0.82) and good-to-excellent accuracy in predicting tibial component size in medial unicompartmental knee replacement. While exact match rates were moderate (51%-61%), clinically acceptable accuracy within ±1 size was achieved in 87%-96% of cases. The high reproducibility across observers supports its use as a reliable surgical planning adjunct. However, systematic sizing bias can occur, highlighting the importance of awareness of radiographic limitations and the significance of intraoperative assessment in determining the final implant size.
